# Usage of Underground Space for 3D Cadastre Purposes and Related Problems in Turkey

**DOI:** 10.3390/s8116972

**Published:** 2008-11-05

**Authors:** Cevdet C. Aydin

**Affiliations:** Hacettepe University, Buklum sokak 74/10 06700 Kavaklı dere, ANKARA, Turkey; E-mail: ceaydin@hacettepe.edu.tr

**Keywords:** 3D cadastre, underground space, registration, legal context

## Abstract

Modern cities have been trying to meet their needs for space by using not only surface structures but also by considering subsurface space use. It is also anticipated that without planning of underground spaces for supporting surface city life in the years and generations to come, there will be serious and unavoidable problems with growing populations. The current Turkish cadastral system, including land right registrations, has been trying to meet users' needs in all aspects since 1924. Today Turkey's national cadastre services are carried out by the General Directorate of Land Titles and Cadastre (TKGM). The Cadastre Law, Number 3402, was approved in 1985 to eliminate problems by gathering all existing cadastral regulations under one law and also to produce 3D cadastral bases to include underground spaces and determine their legal status in Turkey. Although the mandate for 3D cadastre works is described and explained by the laws, until now the bases have been created in 2D and the reality is that legal gaps and deficiencies presently exist in them. In this study, the usage of underground spaces for the current cadastral system in Turkey was briefly evaluated, the concept of 3D cadastral data is examined and the need for using subsurface and 3D cadastre in addition to the traditional 2D register system, related problems and registration are mentioned with specific examples, but without focusing on a specific model.

## 1. Introduction

There has been a growing interest in representing and analyzing the real world using 3D tools. In particular countries such as The Netherlands's, Israel, Greece, Malaysia, Norway, Sweden, Canada, Denmark, etc, use 3D cadastral data registrations [9] in addition to their national 2D bases and at the same time they have also been trying to resolve their fundamental technical and legal issues.

In Turkey, the cadastre system is based on the cadastral works that were carried out during the Ottoman Empire period. The current primarily 2D cadastral system is regularly updated both in rural and urban areas by the Turkish Land Registry and Cadastre Information System project (TAKBİS), which was put into practice by the General Directorate of Land Titles and Cadastre (TKGM) to overcome cadastre based problems with digital technology and GIS capabilities. To date all geometric and attribute information such as geometry and land unit data are considered to be 2D in nature and the cadastre only deals with property located on the surface and its rights.

When a 3D cadastre is mentioned about, people only understand xyz cadastral coordinates instead of thinking about how to use the above surface and underground space, analyzing 3D cadastral registration, modeling 3D objects in data bases, etc. Today, standards are used and 3D coordinates produced in cadastre work. In particular new cadastral databases with 3D coordinates have been produced by the private sector using Turkish National Fundamental GPS Network standards (TUTGA) since 2005, but old cadastre sheets are in 2D and not in digital form. That means that for urban areas which have complex construction and engineering projects, including usage of underground spaces, there are 3D registration problems and the needed 3D data are currently mostly in 2D form.

A similar study about a 3D cadastre was conducted and presented at a FIG Working Week 2004 (Athens, Greece) [1]. In spite of putting too much stress on 3D cadastre and its basic components 3D geo-information, cadastral bases, maps and attribute data have not been produced and prepared in 3D format, especially in urban areas in Turkey. The usage of underground space has been a necessity until now, but today it is a land use type. Urban people and decision makers want to evaluate underground space for efficient land use.

Also the third dimension is a source of commercial income and a criterion which increases the land value. One of the important questions is the intended usage of under and above surface space. Therefore it is urgent to form these 3D bases for constructing economic, rapid and accurate big engineering projects carried out especially in big cities in Turkey like Istanbul, İzmir, Ankara and Konya. etc. Decision makers in urban developments are trying to find new solutions for land use problems by intending to use above and underground space. Because the necessary bases, data and legal arrangements are unavailable, many application and registration problems have occurred in big cities in Turkey. 3D Land object properties should be included in the traditional 2D cadastral system both technically and legally when title deed information is transferred to the database. It is easy to collect these types of data like spatial, register, vertical, title deed information and images of land objects etc. in any coordinate system and position accuracies with today's technologies. All that remains is to include these types of 3D data into the existing cadastral system.

The objective of this paper is to investigate the 3D cadastre concept under Turkey's conditions. The history of the legal context, current situation, the need for 3D cadastre data, the importance of object registration in 3D form, usage of under and above surface objects and relevant problems were all studied.

## 2. The Current System

### 2.1. History of the Legal Context

The Republic of Turkey was founded in 1923 and cadastral works were started in 1924 with the law numbered 474. This law provided for the determining real estates' owners, incomes, values and geometrical situations in some counties and provinces. The cadastre organization was constituted under the General Directorate of Land Registry in 1925 under law No. 658. The purpose of this organization was determining property and boundaries of real estates and their classification in terms of position and economic situation. Then cadastral work was started in some major cities. In 1934, the Cadastre and Land Registry Law was put into practice and regulations were prepared in 1935. Cadastral works were carried out with respect to this law especially in urban areas. In 1950, the Land Registry Law numbered 5602 was put into practice to speed up cadastral work in rural areas. Known as ‘land cadastre’, this law was changed in 1964 and 1966 and became the Land Registry Law (No 766). Cadastral work had been carried out in urban and rural areas under two different laws until 1987. The Cadastre Law Numbered 3402 was put into practice to eliminate the problems originating from having two different laws and to gather all cadastral regulations into one law. However, in the forest areas the cadastral works are still carried out under a different law (Number 6831) undertaken by the General Directorate of Forests [3].

### 2.2. The current situation

Cadastral works have speeded up after the 1950's in Turkey. Today, 97% of the cadastre has been completed in urban areas and about 67% in rural areas. However, different measurement methods (photogrammetric, orthogonal, tachometric and graphic), different coordinate systems and scales have been used in these works. The most common method is the graphic one and 20% of all the cadastral maps in Turkey have been produced by this method, without a coordinate system. In addition, only 29% of the produced cadastral maps are in digital form, which is a basic requirement for land related information systems. Cadastral maps have also been produced with different coordinate systems. Some of them are in local coordinate systems and the others are in a national coordinate system (Table 1).

Because of these factors, it is very difficult to form digital cadastral maps from these bases. Although different studies have been carried out since the 1990's to digitize these maps with the required accuracy, the results have not been as expected. Furthermore, the existing legal structure is not give permission to renew the cadastre and nowadays the need for renewing the cadastre has become a pressing current.

**Figure 1.**
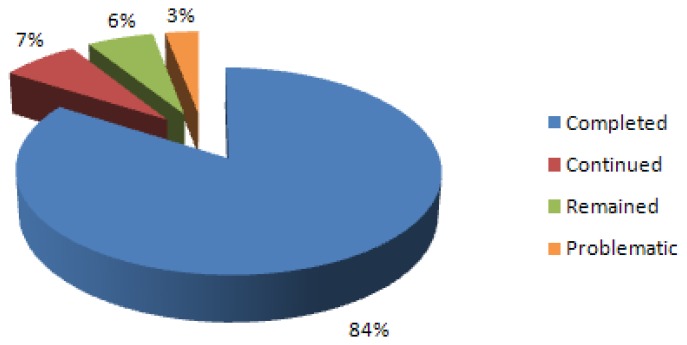
Latest situation of cadastre works in Turkey.

All these reasons indicate that first a legal arrangement is necessary. Secondly, a new cadastral concept should be formed in a multipurpose cadastre context. It maybe includes gathering land use related data in a re-cadastre [4]. After 2005, private sector was involved in cadastre works and according to the TKGM official website [7], 84% of cadastre works have been finished and rest of cadastre will be completed by the end of 2008 (Figure 1).

### 2.3. TAKBIS

**Figure 2.**
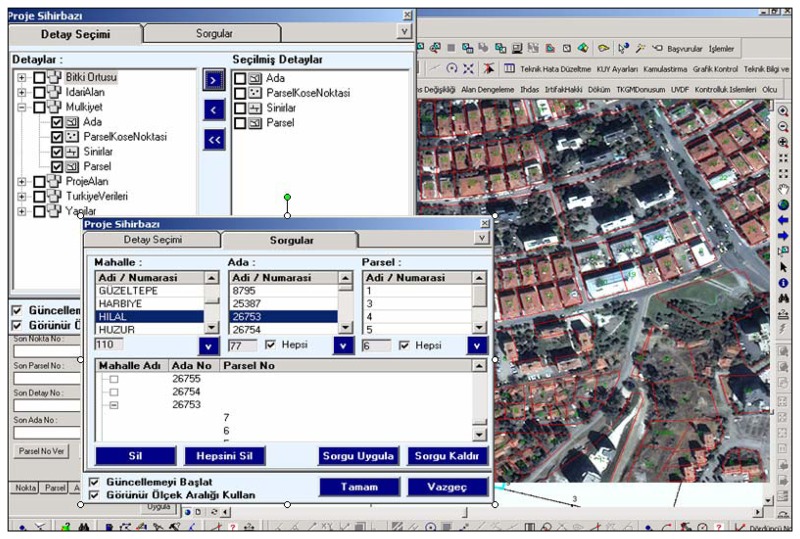
Land parcels, data base integration and related information (geometric and attribute) with the combination of aerial photograph and cadastral map.

As a result of developing technology, some important studies were started in 1980's to achieve computerization of the cadastral works by TKGM. The project called is TAKBİS and was started in 1990's. The goal of TAKBIS is to establish a country-wide land information system (LIS) through the use of GIS and to develop several GIS-based applications, using software required by the end-users (Figure 2).

The initial project investment was estimated at 150 million USD. The first phase of the project, which included a pilot project area in Ankara, involved 360,000 land units with over 1 million land ownership documents. These have been integrated with spatial information systems, taking a year to complete. The project is still under development, and consists of three steps: analysis, design, and application development. The main purposes of TAKBIS are:

- To provide reliable land information required for land and land-related activities and decision makers
- To regulate such activities in accordance to the principles of GIS/LIS in frame of standards of OpenGIS Consortium
- To maintain information updated and re-evaluating them within the scope of information technologies
- To provide spatial data for use in central and provincial public organizations [10].

### 2.4. Data Bases and Registration

The cadastral geometric database includes digital maps containing survey accurate data such as coordinates of boundary points and provides vital land information such as parcel location as well as its ownership (Figure 2). A cadastral registration consists of an administrative and a spatial part mostly maintained in databases. In this way, the modern land registration which is anticipated in Turkish Civil Code will be founded. All cadastral work which are being maintained in order to establish a modern land registration system is done according to a development plan and under the responsibility of the state.

The cadastral registration is a legal record of the rights that are registered on land. Traditionally the cadastre information in Turkey is defined in 2D. The objects are digitally stored and maintained in spatial information systems. Parcels and objects legal status are registered in two dimensional forms. The cadastral system has all CAD capabilities to be able to generate 3D registration and all relations between geometric and attribute data.

## 3. Need for 3D Cadastre in General for Turkey

There has been a considerable increase in population density during the last 30 years in Turkey making land use more intensive, especially in big cities and on valued properties. Today, because of immigration, urban areas have been complex topics for land use.

The relationship of the cities objects such as; buildings, underground tube stations and traffic constructions, etc. and many other building changes are possible to realize using a 3D cadastre. This important step should be supported to some criteria such as intended use and value (Table 2). Data retrieval and data access of information systems of cities need to deal with this complexity. It is clear that we will have excellent tools and powerful systems for the next years to manage 3D large scale databases and to meet the requirements.

In Turkey, some serious problems have been occurring during the construction stage of multipurpose engineering projects because of the lack of vertical information. Especially in the last 20 years, much multipurpose construction projects have been carried out, such as underground tube and utilities tunnels works, rehabilitation of electricity cable works and re-establishment of clean water pipeline works. An example of these problems took place on Friday, August 11, 2006 in the Mecidiyeköy district in Istanbul, when during underground station construction and because of lack of knowledge about existing underground objects on the construction line, a company who was excavating the foundation for a new building on the site had reached a depth of 30 meters, and had even penetrated the 60-centimeter-thick security wall of the Metro. The municipality established a crisis center immediately after the accident. It said the investigation had revealed that there were no plans and maps showing the metro stations underneath. It was noted that the train that hit the pipes was carrying 700 passengers at the time of the accident. Two carriages were damaged in the collision and damage cost 100 thousand Euros [8]. Also at the same area, some apartment's underground parking lots were destroyed and some technical and legal problems appeared and are still ongoing. People also want to have the legal status of their property clearly defined in the cadastre. That gives a clear and serious responsibility to cadastre defines the boundaries of property in all dimensions. Otherwise it is possible to have same problems in future.

## 4. Example of 3D Property Registration and the Legal Context in Turkey

The presentation of 3D environment with 3D structural data, and maintenance of all activities with capable data bases is the modern issue on the agenda for urban data management. Researches and scientific related activities take place all over the world in order to support this important step in the evaluation of urban data management systems and can solve the problems of description and definition of properties, especially in metropolitan areas. The problem is how to register overlapping and interlocking constructions when projected on the surface in a cadastral registration that registers information on 2D parcels. Although properties have been located on top of each other for many years, it is only recently that the question has been raised as to whether cadastral registration should be extended into the third dimension [6].

The public services, investors and real estate developers have been trying to solve these problems above and beneath the surface in Turkey. The legal problem part of the 3D cadastre has been solved by establishment of right of easement, but there are many problems faced in practice. Today these problems are solved using the above rights and upper right in law by relevant institutions to negotiate the matters. So the legal gaps can be easily found in registration of third dimension [1]. Some examples are given below from cn correspondence with TKGM and its organizations.

**Figure 3.**
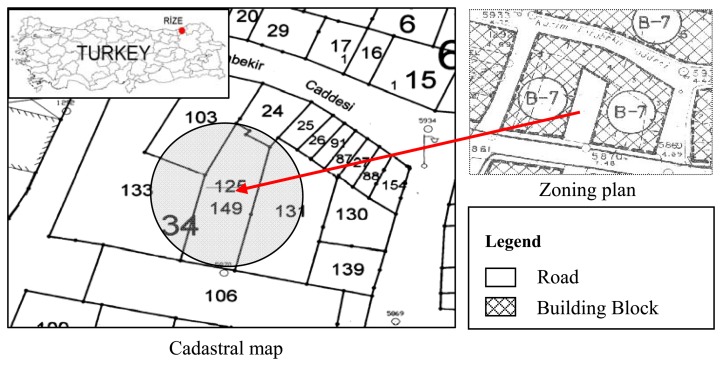
A shop built under parcel 149 in Çarşı district with zoning plan.

“A shop built under parcel 125 (was changed after being registered to 149); block 34 in Çarşı district, Rize province of Turkey (Figure 3). The land including the shop underneath will be registered as a road attribute to Rize Municipality and legal documents will be arranged in the name of the shop's owners according to Civil Law section 652 or 751. Also the surface right will be established and this procedure will be done under direction of notice 1508 (Number: 1001-1525/9-1331, Subject: Registration, Date: 5 April 1991)”.

**Figure 4.**
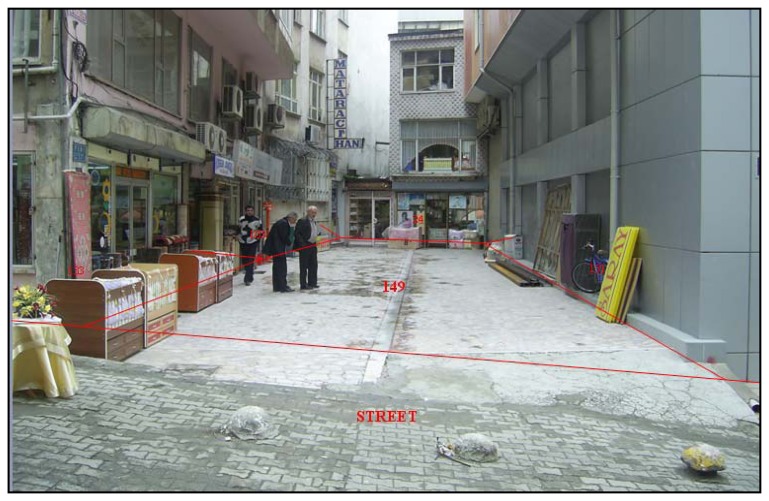
Parcel 149 was formed by excavating from underneath parcel 131.

As described in the correspondence above, according to Cadastre law 3402/4, a shop was built under parcel 149 in block 34 in Çarşı district, Rize province of Turkey As shown in Figure 4, the shop was formed by excavating from underneath parcel 131. The land (parcel 125) was then registered as a road in the name of Rize Municipality and rented to someone for 49 years. After registration the parcel number was changed to 149. So the upper right was established on parcel 149 and a registration page was prepared.

**Figure 5.**
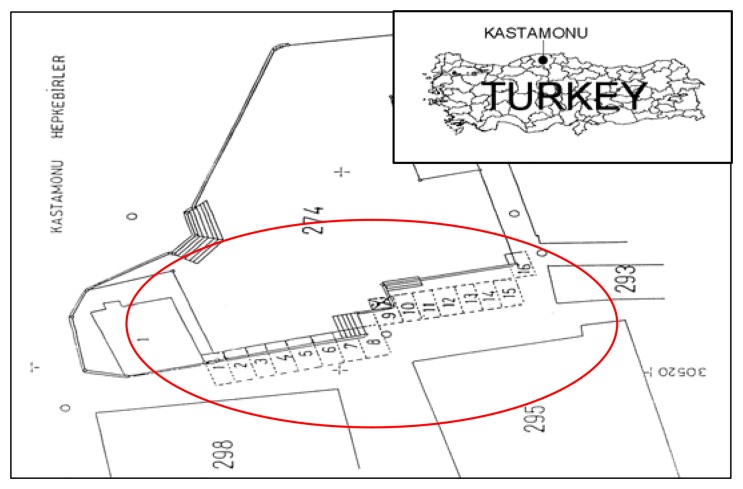
Description of shops built under Mutaflar Street.

Another example is some shops built under Mutaflar Street by Mutaflar municipality in the Kastamonu province of Turkey (Figure 5).

The roads within municipality borders belong to the municipality. It is possible to register the roads above the shops in the name of Hepkebirler municipality and shops can be registered as individual and permanent rights according to civil law section 826. (Number: 1001-1525/4-1684, Subject: Access right, Date: April 13 1990).

**Figure 6.**
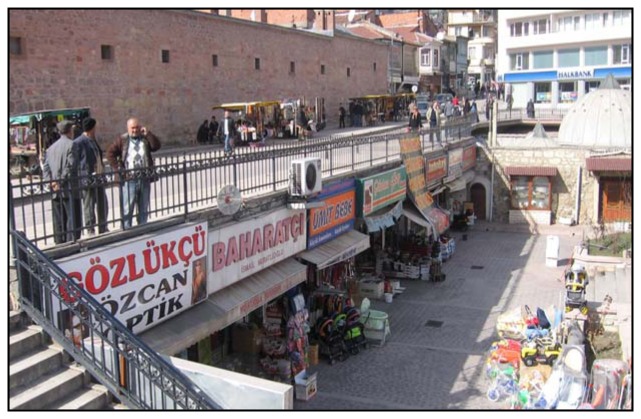
Shops underneath the road in Hepbekirler municipality.

As shown with striped lines in Figure 6, some shops were constructed under Mutaflar Street. The land part (parcel 274) containing the shops underneath was registered as a road attribute in the name of Kastamonu Municipality and shops were registered as a right of easement.

As mentioned above, problems have arisen from been trying to use the “upper right” concept defined as building buildings on or underneath a land whose property right belongs to someone else (Turkish Civil Law / 751) in Turkey. According to the civil law: “if someone has the right of ownership that means has the under and above rights, etc.” Another way of saying is the owner has the use of under and above its parcel. On the other hand 652 section of civil law says “under and above rights of any properties can be belong to someone else with registration to title deeds as a right of easement”. So, legal gaps and confusions can be easily founded in registration of the third dimension [1].

The most important cadastral registration is the right of ownership. The right of ownership is established on a parcel and applies for all space above and below the surface parcel, i.e. the ownership of a parcel is not limited in the third dimension. An owner of a parcel can be restricted in using the whole parcel column by establishing limited real rights on the parcel, by establishing apartment rights or by imposing Public Law restrictions [6]. In Turkey “upper right” is defined as a kind of right of easement in Turkish civil law. Especially underground shopping centers registrations are made in practice with right of easement. Another problem is which criterions will be taken into consideration to establish upper right on someone else's parcel.

## 5. Evaluating 3D Cadastral Objects in Turkey

Although the current cadastral data bases have the ability of registering the objects in 3D forms they are registered in 2D. Factual situations arise for which it has become apparent that the current 2D system is not able to represent the legal status of the situation in the most efficient manner. In this section some examples will be given about 3D cadastre subjects relevant to objects such as underground stations, multilevel underground car parks, etc. unseen in 2D digital photogrammetric maps and which can be presented in 3D Geographical Information Systems (3DGIS) [2]. 3DGIS with its tools and modeling techniques manage the large amount of property data related underground and above spaces, produce more accurate results for 3D volumetric calculations, and support a complete understanding of objects and geometric variations in the space by the real time interactive procedures [5].

### 5.1. Underground Shopping Centers and Underground Parking Lots

Underground shopping centers are the objects unseen on air photographs. They remain under the surface when photographs are digitized and are used as commerce and relaxation centers that serve a wide population. These objects can be originally presented and registered in 3D data. An example is Aksaray square underground shopping centre under Ordu Street in Istanbul, which looks like a small district with its own management department, workers, shops, infrastructures, etc.

The centre shelters a pharmacy, an optician, a perfumery, a jeweler, a sweet shop, a WC, a tea house, a change office and 134 work shops. The shops have surface areas ranging from between 16 m^2^ to 219 m^2^. And the centre has a manager, a secretary, a technician, 10 security officials and three cleaning officials, for a total of total 15 people on duty.

**Figure 7.**
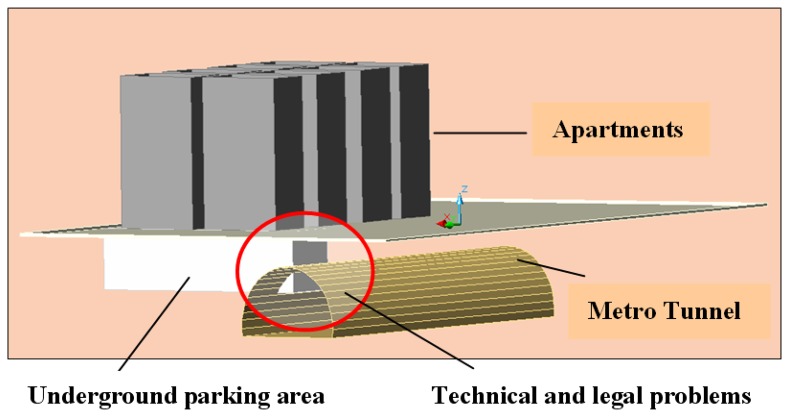
Apartments with underground parking lot problems.

Also underground parking lots are frequently used in the urbanization process; generally the lower level of buildings is using as an underground parking space in Turkey. These areas are sometimes larger than the building based area and is not seen on maps. The Aksaray underground parking lot, for example, has three entry-exits and a capacity of 350- 400 cars and is under Turgut Özal Street in Aksaray Square in Istanbul. In Turkey and all over the world so many problems have occurred at the construction stages. The workers can be faced up some overlap problems because of not having necessary and updated geometric data located on maps especially during excavations works inside municipality's borders in Turkey (Figure 7).

Another legal problem related with the topic is the right of easement of the parcels under which which metro lines pass through. Owners go to court for indemnity payment because of metro line use their parcels underneath and the courts having difficulties in making a decision due to legal gaps. The point is whether metro line restricts the usage of a parcel. If there are no restrictions does that mean no indemnity payments are due? Another question is how many meters does the metro line pass through underneath a parcel.

Eventually, underground constructions have been carried out. During the stage of opening underground canals some overlapping troubles have occurred due to lack of up-to-data geometric information. So these situations are the other examples of the necessity for 3D cadastre bases.

### 5.2. Underground Stations

Underground tube stations are one of the important components for daily life. They shelter a lot of service, shopping and rest structures and have their own settlement plans, security systems, management systems etc. Briefly they are small models of the cities and unseen on the maps. The Kızılay underground station in the Turkish capital Ankara is an example which looks like small units of the city. It has a large data density for planning a GIS project and is a very good example of using the underground parcels for 3D cadastre. As mentioned above, these objects can be presented in 2D and 3D forms in systems

### 5.3. Pedestrian Subways

Pedestrian subways have the same characteristics as the objects mentioned above. They serve daily life with their commercial structures and pedestrian density. Haşim İşcan Subway in Istanbul is one of the important transport centers and the main place for the toy sector [2].

The examples in this chapter underline once again the need for more information in the case of 3D situations. A lot of records can be found in data bases indicating a 3D situation and all these situations express that the third dimension is relevant in confirming the legal status of the real estate objects. At present, apart from apartments, there are no formal rules for registering the legal status, geometric location and extent of 3D objects.

## 6. Conclusions

The current Turkish Cadastral System includes the TAKBİS organization which is capable of registering complex situations and has performed its tasks for surveying properties, defining their legal status and obtaining the necessary information in 2D form. In the last ten years some new needs have been occurring concerning multilevel land use in Turkey. All these developments of land use have brought the problem of registration in complex situations together.

There are many complex situations related to 3D cadastre waiting for solutions and the numbers of them are increasing. A conceptual model that includes legal and technical details should be developed to overcome these problems. Another point which must be revised is the legal concept of land use in Turkey as some of the concepts in use come from traditional ways and should be evaluated with today's necessities in mind.

The laws establish the legal status concerning uses above the surface in Turkey, but there are no limitations and restriction for under surface space. Because of the definition the certain rights about under and above surface areas, some restrictions about under surfaces must be considered and shown on zoning plans. I have concluded that current system has all abilities to sort all these problems out with necessary legal arrangements using 3DGIS capabilities. Turkey should be ready for 3D registration for national cadastre on its way to membership in the European Community.

## Figures and Tables

**Table 1.** Turkish cadastral maps according to scales, production methods and sheet bases [7].

**Num**	**Scale**	**Sheet Number**	**%**	**Production Method**	**Number**	**%**	**Base Type**	**%**

1	1/500	18,174	7	Graphic	110,710	0.20	Transparent	25
2	1/1000	68,443	26	Polar	127,118	0.24	Aluminum	31
3	1/2000	93,663	35	Orthogonal	61,271	0.11	Paper	44
4	1/2500	16,877	6	Photogrammetric	87,254	0.16		
5	1/5000	66,218	25	Digital	154,008	0.29		
6	Others	1,329	1					

Total		264,707	100		540,361	100		100

**Table 2.** The usage of underground and its surface structures according to their intended use.

**Under surface**	**Subsurface**

Tube stations	Park, road, building, trade
Shopping centers	Road, pavement, park, shops
Pedestrian subways	Park, road
Parking lots	Building, garden
Bus/tram/railway stations	Offices and shops
Infrastructure Objects: Electricity,	Roads, building complexes,
water, communication, cables, pipelines, sewers, etc.	shops etc.
